# Comparative study on electrocardiograms and serological examinations of acute pulmonary embolism and acute non‐ST elevation myocardial infarction

**DOI:** 10.1111/anec.12920

**Published:** 2021-12-09

**Authors:** Zhihong He, Wenmiao Bi, Zhe Lang, Yanjie Zhen, Ying Jin, Hongjuan Liu, Dongfu Li, Xiaoning Hu, Huanling Li

**Affiliations:** ^1^ Department of Emergency Medicine Shijiazhuang People's Hospital Shijiazhuang China; ^2^ Department of Respiratory Medicine Shijiazhuang People's Hospital Shijiazhuang China; ^3^ Shijiazhuang Community Health Service Management Center Shijiazhuang China

**Keywords:** acute non‐ST elevation myocardial infarction, acute pulmonary embolism, D‐dimer, electrocardiogram, myocardial zymogram, troponin

## Abstract

**Background:**

The aim of this study was to investigate the value of electrocardiograms (ECGs) and serological examinations in the differential diagnosis of acute pulmonary embolism (APE) and acute non‐ST elevation myocardial infarction (NSTEMI) in order to reduce the rate of clinical misdiagnosis.

**Methods:**

The clinical data of 37 patients with APE and 103 patients with NSTEMI admitted to our hospital were retrospectively analyzed. The differences in the clinical manifestations, ECGs, myocardial zymograms, D‐dimers, and troponin (cTn) of the two groups were compared.

**Results:**

In the patients with APE, the main symptom—found in 25 cases (67.56%)—was dyspnea, while in the patients with NSTEMI, the main symptom—found in 52 cases (50.49%)—was chest tightness. The incidences of sinus tachycardia and S_I_Q_III_T_III_ in the group of patients with APE were higher than in the group of patients with NSTEMI, and the difference was statistically significant (*p* < .05). There was no statistical significance in the difference of aspartate aminotransferase and lactate dehydrogenase (LDH) in the two groups (*p* > .05), although there was a statistically significant difference of creatine kinase (CK) and the creatine kinase isoenzyme‐MB (CK‐MB) in the two groups (*p* < .05). The levels of D‐dimers and cTn were increased in both groups, but the level of D‐dimers in the group of patients with APE was higher than that in the group of patients with NSTEMI.

**Conclusion:**

With the occurrence of clinical manifestations like dyspnea, chest tightness, chest pain, and palpitation of unknown causes, the possibility of APE and NSTEMI should be considered.

## 1 BACKGROUND

Acute pulmonary embolism (APE) is one of the most common diseases seen in the emergency department. The recent prevalence of APE has increased year on year. Due to its acute onset and lack of specific symptoms, APE has a high rate of misdiagnosis and mortality. If not treated properly, it is likely to develop into chronic pulmonary embolism and may be fatal: about 300,000 people die of APE every year (Howard, 2019). APE is a serious social health problem that affects the safety and quality of life of patients (Daquarti et al., 2016). Its main clinical manifestations are chest pain and dyspnea, and it is easily misdiagnosed as acute coronary syndrome (ACS), especially in the case of acute non‐ST elevation myocardial infarction (NSTEMI). Therefore, early and accurate diagnosis is particularly important for the treatment and prognosis of patients with APE. Electrocardiograms (ECGs) and serological examinations are the routine indexes to differentiate APE from ACS after admission. In the present study, the ECGs and myocardial zymograms of 37 patients diagnosed with APE in our hospital, and 103 patients with NSTEMI from the same period, were compared. The aim of the study is to reduce the rate of misdiagnosis of the two diseases and improve diagnostic accuracy.

## 2 METHODS

### 2.1 Study sample

A total of 140 patients admitted to our hospital from January 2015 to December 2018, of which 37 had been diagnosed with APE and 103 with NSTEMI, were retrospectively selected. The diagnosis of APE was in accordance with the relevant diagnostic criteria in the guidelines for the diagnosis and treatment of pulmonary thromboembolism formulated by the respiratory branch of the Chinese Medical Association (Chinese society of respiratory diseases, 2001), and was confirmed by pulmonary CTA and pulmonary angiography. The diagnosis of NSTEMI met the relevant diagnostic criteria in the guidelines for the diagnosis and treatment of NSTEMI (Chinese Society of Cardiology, ). Of the 103 patients with NSTEMI, 90 were diagnosed using coronary angiography.

### 2.2 Materials and methods

The clinical manifestations, ECGs, myocardial zymograms, D‐dimers, and cardiac troponin (cTn) in patients with APE and NSTEMI were compared and analyzed. ECG examinations were performed with 12 leads; the paper speed was 25 mm/s and the standard voltage was 10 mv. The specialist in the ECG department had the responsibility of making the final decision on the controversial ECG manifestations. The myocardial zymograms were obtained from blood samples taken from the elbow vein at admission. The instrument used was the Beckman AU5800 biochemical analyzer. D‐dimers and cTn were determined using chemiluminescence. Beckman ACCESS 2 was used as the detection instrument.

### 2.3 Statistical analysis

SPSS 19.0 software was used for statistical analysis. A Chi‐squared test was used to count data, and *p* < .05 was considered statistically significant.

## 3 RESULTS

### 3.1 General characteristics

Among the 37 patients with APE, 20 (54.1%) were male and 17 (49.9%) were female. Their ages ranged between 16 and 96, with the average age being 63 ± 6.66 years. Among the 103 patients with NSTEMI, 55 were male (53.40%) and 48 were female (46.60%). Their ages ranged between 45 and 86, with the average age being 64 ± 7.81 years. There was no significant difference in the gender composition or age distribution of the two groups. See Table 1.

**TABLE 1:** Comparison of general characteristics between the two groups

Item	*N*	Male	Female	Age (Year, $\bar{x}\pm s$)
APE	37	20 (54.10%)	17 (49.90%)	63 ± 8.32
NSTEMI	103	55 (53.40%)	48 (46.60%)	65 ± 12.55
Statistical value			χ^2^ = 0.005	*F *= 3.301
*p*			*p *> .05	*p *> .05

### 3.2 Basic diseases

The main basic diseases of the two groups were hypertension, cerebrovascular disease, hyperlipidemia, diabetes, respiratory disease, digestive system disease, other endocrine disorders, and deep varicose veins of the lower extremities. Although there were many basic diseases in both groups, the incidence of varicose veins in the APE group was significantly higher than that in the NSTEMI group and the difference was statistically significant (*p* < .05). There was no significant difference in the incidence of other basic diseases between the two groups (*p* > .05). See Table 2.

**TABLE 2:** Comparison of basic disease between the two groups (*n*, %)

Basic disease	APE	NSTEMI	*p*
Hypertension	20 (54.05%)	55 (53.40%)	.945
Cerebrovascular disease	7 (18.92%)	27 (26.21%)	.375
Hyperlipidemia	8 (21.62%)	32 (31.07%)	.275
Diabetes mellitus	5 (13.51%)	21 (20.39%)	.356
Respiratory disease	6 (16.22%)	10 (9.71%)	.286
Digestive disease	5 (13.51%)	20 (19.42%)	.421
Disease of other endocrine systems	2 (5.41%)	9 (8.74%)	.772
Surgery	7 (18.92%)	14 (13.59%)	.436
Deep varicose veins of the lower extremities	14 (37.84%)	5 (4.85%)	.001
Healthy	4 (10.81%)	23 (22.33%)	.128

### 3.3 Clinical manifestations

**FIGURE 1:**
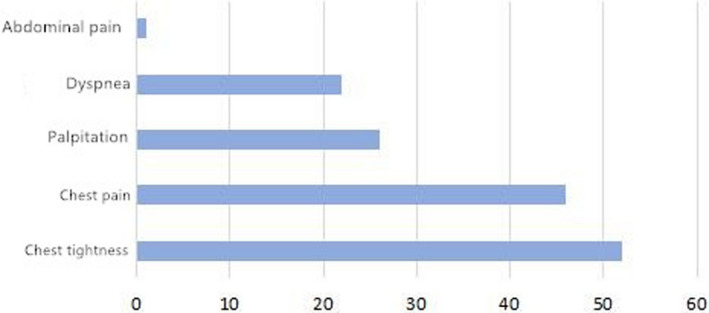
Clinical manifestations in 37 patients with APE

**FIGURE 2:**
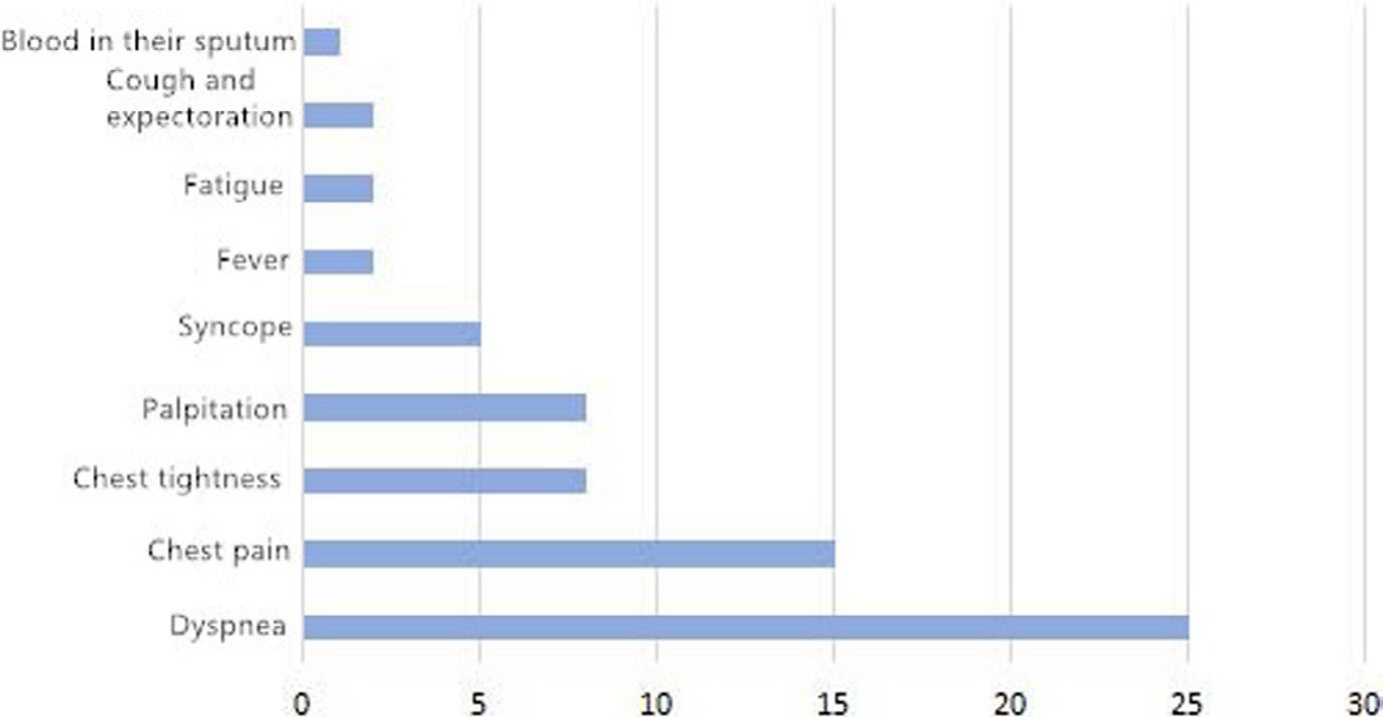
Clinical manifestations in 103 patients with NSTEMI

Among the 37 patients with APE, 25 (67.56%) had dyspnea, 15 (40.54%) had chest pain, 8 (21.62%) had chest tightness, 8 (21.62%) had palpitations, 5 (13.51%) had syncope, 2 (5.41%) had fever, 2 (5.41%) had fatigue, 2 (5.41%) had a cough and expectoration, and 1 (2.70%) had blood in their sputum. Among the 103 patients with NSTEMI, 52 (50.49%) had chest tightness, 46 (44.66%) had chest pain, 26 (25.24%) had palpitations, 22 (21.36%) had dyspnea, and 1 (0.97%) had abdominal pain. The details are illustrated in Figures 1 and 2.

### 3.4 Changes in ECGs

The incidence of sinus tachycardia and S_I_Q_III_T_III_ in the group of patients with APE was higher than in the group of patients with NSTEMI, and the difference was statistically significant (*p* < .05). There were no significant differences between the two groups in premature ventricular contractions, left and right bundle branch blocks, ST‐T changes in leads II, III, and AVF, ST‐segment depression in the precordial leads, and T‐wave inversion in the precordial leads (*p* > .05). See Table 3.

**TABLE 3:** Comparison of ECG between the APE group and the NSTEMI group [Case (%)]

ECG manifestation	APE group	NSTEMI group	χ^2^	*p*
Sinus tachycardia	25 (67.57)	24 (23.30)	23.447	.001
Right bundle branch block	7 (18.92)	9 (8.74)	2.787	.095
Left bundle branch block	0 (0)	10 (9.71)	3.869	.063
Ventricular premature beat	4 (10.81)	8 (7.77)	0.322	.571
S_I_Q_III_T_III_	7 (18.92)	0 (0)	20.512	.001
ST‐T changes in leads II, III, and AVF	5 (13.51)	17 (16.50)	0.184	.668
Precordial ST‐segment depression	12 (32.43)	37 (35.92)	0.146	.703
T‐wave inversion in precordial lead	14 (37.84)	39 (37.86)	0.000	.998

### 3.5 Results of the myocardial zymograms

A myocardial zymogram is composed of aspartate aminotransferase (AST), creatine kinase (CK), creatine kinase isoenzyme‐MB (CK‐MB), and lactate dehydrogenase (LDH). There was no significant difference in AST or LDH between the two groups of patients (*p* > .05), but there was significant difference in CK and CK‐MB between the two groups (*p* < .05). See Table 4.

**TABLE 4:** Comparison of the results of myocardial zymogram between the APE group and the NSTEMI group [Case (%)]

Myocardial zymogram	APE	NSTEMI	χ^2^	*p*
CK	5 (13.51)	89 (86.41)	65.564	<.01
CK‐MB	6 (16.22)	72 (69.90)	31.799	<.01
LDH	21 (56.76)	75 (72.82)	3.257	.071
AST	26 (70.27)	87 (84.47)	3.524	.06

### 3.6 D‐dimer and cTn results and the ratio of D‐dimer/cTn (Rd)

The level of D‐dimers in the group of patients with APE was higher in the group of patients with NSTEMI, and the difference was statistically significant (*p* < .05). The level of cTn in the patients with APE was lower than in the patients with NSTEMI (*p* < .05), and the Rd in the patients with APE was higher than in those with NSTEMI. See Table 5.

**TABLE 5:** Comparison of D‐dimer, cTn, and Rd between the two groups

Grouping	D‐dimer (μg/ml)	cTn (ng/ml)	Rd
APE	2.539 (0.794,3.998)	0.020 (0.010,0.070)	91.112 (18.962,215.526)
NSTEMI	0.344 (0.293,0.402)	1.906 (0.522,6.129)	0.179 (0.060,0.683)
χ^2^	39.949	39.958	39.945
*p*	*p *< .05	*p *< .05	*p *< .05

## 4 DISCUSSION

APE and NSTEMI are both emergencies with high mortality. They manifest as chest pain, shortness of breath, and dyspnea. As such, it is difficult to diagnose and differentiate between APE and NSTEMI based on the clinical symptoms alone, which may lead to missed diagnosis and misdiagnosis (Research group of standardized diagnosis & treatment of pulmonary embolism, 2006). However, due to the different treatment schemes of APE and NSTEMI, it is of great clinical significance to differentiate the two. In recent years, pulmonary angiography has become the “gold standard” for the diagnosis of APE. However, due to limitations like its high cost and invasive nature, clinical application is greatly limited (Ghatak et al., 2015). As ECGs are simple, economic, and effective in application, they have instead become the first choice in differential diagnosis. Combined with changes in the levels of myocardial enzymes, ECGs have certain reference significance in differential diagnosis.

In terms of the clinical manifestations of the two diseases, the results of the present study show that APE and NSTEMI can manifest as dyspnea, chest pain, chest tightness, and palpitation. In the group of patients with APE, the main symptom was dyspnea, which was found in 25 cases (67.56%), followed by chest pain, which was found in 15 cases (40.54%). In the group of patients with NSTEMI, the main symptoms were chest tightness (50.49%), which was found in 52 cases, and chest pain, which was found in 46 cases (44.66%). As such, it was suggested that the occurrence of dyspnea was more prominent in patients with APE. It was also found that 14 patients in the APE group and 5 in the NSTEMI group had deep varicose veins of the lower extremities, and the difference was statistically significant. The result indicates that patients might be at risk of deep venous thrombosis (DVT) of the lower extremities. DVT is a high‐risk factor for pulmonary embolism, and its existence might provide some clues as to the differential diagnosis between the two diseases.

Concerning the ECG manifestations of the two diseases, some studies have found that the main ECG characteristics in patients with APE are the pulmonary P wave, right bundle branch block, and right axis deviation, while the main characteristics in patients with NSTEMI are T‐wave inversion and ST‐segment depression or elevation. In the present study, ECG manifestations in the group of patients with APE and the group with NSTEMI were compared. It was found that the incidence of sinus tachycardia and S_I_Q_III_T_III_ in the group of patients with APE was higher than in the group with NSTEMI and that the difference was statistically significant (*p* < .05). There were no significant differences (*p *> .05) between the group with APE and the group with NSTEMI in the premature ventricular contractions, left and right bundle branch block, ST‐T changes in leads II, III, and AVF, and the ST‐T segment depression in the precordial leads. These results indicate that S_I_Q_III_T_III_ might have a certain value in the diagnosis of pulmonary embolism, but it was not significant enough to distinguish between APE and NSTEMI using the ST‐T changes and right bundle branch block.

This study also found that sinus tachycardia was the most common ECG change in the group of patients with APE (67.57%), followed by T‐wave inversion (37.84%), ST‐segment depression (32.43%), and S_I_Q_III_T_III_ (18.92%). There are three possible main reasons for this. Firstly, due to the blockage of the pulmonary artery, the blood circulation of the pulmonary vascular bed is reduced and the pulmonary artery pressure is significantly increased, which increases the right heart power and oxygen consumption. At the same time, the imbalance of the ventilation/blood flow ratio leads to hypoxemia. In order to maintain the normal metabolism of body tissues, the heart rate is reflexively accelerated. In addition, dyspnea, irritability, and other symptoms can lead to the excitation of the sympathetic nerve. Secondly, with the gradual increase of right ventricular pressure, the pressure difference between the aorta and right ventricle during the systolic period decreases, which causes a decrease in the perfusion pressure of the right coronary artery, the blood flow of the right coronary artery, and the blood supply of the right ventricular myocardium, especially the subendocardial myocardium of the right ventricle. As a result, the ECG manifests ischemic changes that are similar to coronary T changes (Zhong‐qun et al., 2014). One study has suggested that T‐wave inversion could be an indicator of APE and that the severity of the disease might increase with the range of T‐wave inversion moving to the left (Han & Gao, 2017). Thirdly, S_I_Q_III_T_III_ is a common ECG manifestation of pulmonary embolism that is correlated with the rapid expansion of the right ventricle and atrium due to the sharp rise of pulmonary circulation resistance and the increase of pulmonary artery pressure during the acute onset of disease (Ho et al., 2015; Ling et al., 2015). However, in the present study, the incidence of S_I_Q_III_T_III_ was not high, which might be correlated with the area of pulmonary embolism. Liu et al. (2010) suggested that the incidence of S_I_Q_III_T_III_ in patients with large‐area APE and sub‐large‐area APE was significantly increased. Therefore, if a change of S_I_Q_III_T_III_ is found in clinical practice, we should be alert to the possibility of large‐scale pulmonary embolism. Among the ECG manifestations in patients with NSTEMI, T‐wave inversion in the precordial lead was the most common (37.86%), followed by ST‐segment depression (35.92%), which was consistent with the results of Cui (2016).

“Myocardial enzyme” is the general term used to refer to many enzymes that exist in the myocardium, including AST, CK, CK‐MB, and LDH. LDH exists in all tissues and cells, although its content is higher in the kidneys. AST is mainly distributed in the myocardium, followed by the liver, which is helpful in the diagnosis of myocardial injury. CK is mainly distributed in the muscle tissue, followed by the myocardial tissue and brain tissue. CK‐MB is widely distributed in the myocardial cells and its content in other tissue—a specific enzyme of the myocardium—is small. Therefore, CK and CK‐MB are of great significance in the detection of myocardial injury and have certain value in the diagnosis of myocardial infarction (Ding & Liu, 2015). In recent years, clinical studies have shown that 30%–50% of patients with APE have secondary myocardial injury, often with varying degrees of myocardial enzyme abnormalities. The degree of myocardial injury can be used as the basis for disease evaluation (Steering, 2012). The results of the present study show that the myocardial zymograms of patients with APE are significantly higher than those of patients with NSTEMI, and that the levels of CK and CK‐MB in patients with APE are significantly higher than in patients with NSTEMI. Therefore, CK and CK‐MB might be of great value in distinguishing APE from NSTEMI, a finding that is consistent with the results of Zhang, (2016).

An increase in cTn mainly reflects an injury to the myocardial cells and has high specificity and sensitivity of the myocardium. It has become the ideal marker of myocardial infarction and the sensitive and specific marker of micro‐myocardial cell injury. Study has shown that pulmonary artery pressure and right ventricular pressure rise sharply in APE due to pulmonary vascular blockage and contraction, which leads to right ventricular dilation, right ventricular myocardial ischemia, and, sometimes, myocardial infarction, resulting in an increase in the level of cTn (Meyer et al., 2000).

D‐dimers are the degradation product of cross‐linked fibrin. They are a specific marker reflecting the function of the coagulation fibrinolysis system. Their value in the diagnosis of pulmonary embolism has been confirmed in clinical practice. One study (Yildirim et al., 2017) found that, with the injury of the vascular endothelial cells in patients with ACS, the release of coagulant and anticoagulant substances increases, which leads to the imbalance of the coagulation and fibrinolysis system, promotes the formation of coronary artery thrombosis, and causes the activation of the fibrinolytic system. Therefore, the specific indicators of the coagulation and fibrinolysis system are of great significance for the diagnosis of ACS. Both APE and NSTEMI may lead to an increase of D‐dimers and cTn, but the degree of this increase may vary. The results of the present study show that the level of D‐dimers in patients with APE is higher than in patients with NSTEMI and that the difference is statistically significant, whereas the level of cTn in patients with APE is lower than in patients with NSTEMI and that the difference is also statistically significant. In addition, the Rd was proposed to better distinguish between the two diseases in the present study. The results showed that the Rd in patients with APE is significantly higher than in patients with NSTEMI.

## 5 CONCLUSION

In conclusion, the differential diagnosis of APE and NSTEMI should be comprehensively analyzed. With the clinical manifestations of dyspnea, chest tightness, chest pain, and palpitation of unknown causes, the results of ECGs, myocardial zymograms, D‐dimers, and cTn should be combined in the etiological analysis. When considering the diagnosis of ACS, the possibility of APE should be taken into account to avoid misdiagnosis and missed diagnosis.

## CONFLICT OF INTEREST

The authors declare that they have no competing interests.

## AUTHOR CONTRIBUTIONS

ZHH, WMB, DFL, and ZL conceived the idea and conceptualized the study. YJZ, YJ, and HJL collected the data. XNH and HLL analyzed the data. ZL and YJZ drafted the manuscript, then ZL and YJZ reviewed the manuscript. All authors read and approved the final draft.

## ETHICS APPROVAL AND CONSENT TO PARTICIPATE

I confirm that I have read the Editorial Policy pages. This study was conducted with approval from the Ethics Committee of Shijiazhuang people's Hospital. This study was conducted in accordance with the declaration of Helsinki. Written informed consent was obtained from all participants.

## CONSENT FOR PUBLICATION

All participants signed a document of informed consent.

## Data Availability

We declare that materials described in the manuscript, including all relevant raw data, will be freely available to any scientist wishing to use them for non‐commercial purposes, without breaching participant confidentiality.
